# Distribution, Diversity and Roles of CRISPR-Cas Systems in Human and Animal Pathogenic Streptococci

**DOI:** 10.3389/fmicb.2022.828031

**Published:** 2022-01-31

**Authors:** Coralie Lemaire, Brice Le Gallou, Philippe Lanotte, Laurent Mereghetti, Adeline Pastuszka

**Affiliations:** ^1^Université de Tours, INRAE, Infectiologie et Santé Publique, BRMF, Tours, France; ^2^Service de Bactériologie-Virologie, Centre Hospitalier Régional Universitaire de Tours, Tours, France

**Keywords:** CRISPR, CRISPR role, CRISPR application, streptococci, pathogenic streptococci

## Abstract

Streptococci form a wide group of bacteria and are involved in both human and animal pathologies. Among pathogenic isolates, differences have been highlighted especially concerning their adaptation and virulence profiles. CRISPR-Cas systems have been identified in bacteria and many streptococci harbor one or more systems, particularly subtypes I-C, II-A, and III-A. Since the demonstration that CRISPR-Cas act as an adaptive immune system in *Streptococcus thermophilus*, a lactic bacteria, the diversity and role of CRISPR-Cas were extended to many germs and functions were enlarged. Among those, the genome editing tool based on the properties of Cas endonucleases is used worldwide, and the recent attribution of the Nobel Prize illustrates the importance of this tool in the scientific world. Another application is CRISPR loci analysis, which allows to easily characterize isolates in order to understand the interactions of bacteria with their environment and visualize species evolution. In this review, we focused on the distribution, diversity and roles of CRISPR-Cas systems in the main pathogenic streptococci.

## Introduction

Streptococci are cocci Gram positive bacteria arranged typically in pairs or chains. They form a large group of bacteria, composed of 103 species (Patel and Gupta, 2018). They are widely distributed and are particularly found at the surface of mucous membranes, like upper respiratory, gastro-intestinal, or genito-urinary tracts of humans and many animals. Streptococci are also present in the environment, soil, plants, food or dairy products (Hardie and Whiley, 1997; Kok and Hutkins, 2018). Some *Streptococcus* species belong to the microbial flora and are considered opportunistic pathogens that can lead to significant health problems in humans or animals (Krzyściak et al., 2013). *Streptococcus pyogenes* (Cunningham, 2000; González-Abad and Alonso Sanz, 2020) and *Streptococcus pneumoniae* (Musher, 1992; Weiser et al., 2018) are more particularly involved in human pathologies and can cause invasive infections. Other streptococci belong specifically to animal microbiota, like *Streptococcus suis*, which is an important cause of various clinical diseases in swine and domestic animals (Staats et al., 1997; Haas and Grenier, 2018). Also, several species are involved in both human and animal pathology, like *Streptococcus agalactiae*, which represents a major human pathogen causing invasive neonatal infections (Raabe and Shane, 2019), after an initial description in bovine mastitis (Nocard and Mollereau, 1887). More recently, it is also a major pathogen in farm fishes with important economic consequences (Mian et al., 2009; Eto et al., 2020). Finally some streptococci are essential as lactic ferments in agro-food industries (Mercenier et al., 1994; Nagaoka, 2019).

Streptococci were initially classified according to their hemolysis on blood agar which can be complete, incomplete or absent (Schottmuller, 1903). Then Rebecca Lancefield classified beta-hemolytic streptococci based on antigenic properties of the polysaccharide C, a carbohydrate present in the bacterial cell wall (Lancefield, 1933). Many improvements were made in the following years and now, with molecular techniques, like 16S rRNA gene sequence analysis or whole genome sequencing, many more precise classifications have emerged. Nevertheless, despite the number of techniques currently available, none provide a perfect distinction between isolates, especially for clinical isolates belonging to the viridans group. However, in routine laboratory practice, bacterial culture identification is easier and faster since the use of mass spectrometry (Carbonnelle et al., 2011). Matrix Assisted Laser Desorption Ionization Time-Of-Flight (MALDI-TOF) is used daily for bacterial identification and gives reliable results thanks to a relatively rich spectral database which compares the bacterial spectrum obtained for each sample. Despite this powerful technology, some gaps exist. This is the case for the *Streptococcus pneumoniae* species which is difficult to differentiate from the *Streptococcus mitis* and *Streptococcus oralis* group because their spectra are very similar (Marín et al., 2017). Additional tests such as the use of optochin discs are therefore required to complete identification.

Over the past 10 years, CRISPR-Cas systems (Clustered Regularly Interspaced Short Palindromic Repeats and CRISPR—associated proteins) have been described in approximately half of bacterial species, including many streptococcal species, which have been model organisms for their study (Barrangou et al., 2007). Indeed, after several years of research, the immune role of these new systems has been elucidated in *Streptococcus thermophilus* (Barrangou et al., 2007; Horvath and Barrangou, 2010). The initial discoveries for these systems date from 1987 with the analysis of the *iap* gene of *Escherichia coli* (Ishino et al., 1987). They described a region composed of short repeated DNA sequences interspaced with variable sequences. The acronym “CRISPR” was finally approved in 2002 (Jansen et al., 2002). CRISPR loci are composed of short DNA repeats called direct repeats (DR) separated by unique variable DNA sequences called spacers. DR are partially palindromic which give them the ability to form a stable secondary structure in order to interact with other proteins (Kunin et al., 2007; Al-Attar et al., 2011). On the other hand, spacers were suspected to have an extra-chromosomic origin as they share sequence homologies with foreign mobile genetic elements (MGE), such as bacteriophages or plasmids (Bolotin et al., 2005; Mojica et al., 2005; Pourcel et al., 2005). At the 5′ end of the CRISPR locus, the leader is an Adenine and Thymine rich region, which contains the transcription promoter of the CRISPR locus. Generally close to the CRISPR locus, *cas* operon encodes proteins involved in the system’s functionality. The *cas* gene composition is different between systems and gives rise to complex classification. Currently, CRISPR-Cas systems are separated into two classes, six types and 33 subtypes (Makarova et al., 2020). Class 1 systems possess effector modules composed of multiple Cas proteins, while class 2 systems carry a single, multidomain crRNA-binding protein (CRISPR-RNA binding protein) like Cas9, the signature protein of type II systems. Indeed, all systems contain two conserved proteins, Cas1 and Cas2, and a signature protein which is specific of a type.

CRISPR-Cas systems operate in three stages, involving different actors depending on the type of system. During the first stage, called adaptation, a small piece of DNA from a foreign MGE is integrated as a new spacer at the leader end of the CRISPR locus. The second stage, called expression, corresponds to the transcription of the whole CRISPR array into a pre-crRNA, which will be processed subsequently, by many actors, in mature crRNA. A crRNA is composed of a spacer and a part of the adjacent direct repeat (Brouns et al., 2008). During the last stage, called interference, crRNA guides Cas nucleases effector complex toward complementary foreign nucleic acids. After specific recognition, which frequently involves the protospacer adjacent motif (PAM; Mojica et al., 2009), Cas nucleases lead to invader DNA degradation (Garneau et al., 2010; Gasiunas et al., 2012; Sternberg et al., 2014). The sequence homology between spacer and MGE allows the system to fight against MGEs already encountered (Garneau et al., 2010), which is a very particular and innovative mechanism. The recent discoveries enabling us to understand these systems’ mechanisms, and particularly their simplicity of action, have inspired researchers to develop new genetic tools. Today, CRISPR-Cas9 (derived from *S. pyogenes* type II-A system) is internationally used in genetic engineering for genome editing (Jiang et al., 2013). This tool allows to edit genome easily by introducing mutations or deletions in DNA sequences at very specific positions, and thus appears very promising for gene therapy (Hsu et al., 2013; Gori et al., 2015; Broeders et al., 2020) as shown by the recent attribution of the Nobel Prize to Emmanuelle Charpentier and Jennifer Doudna in 2020.

CRISPR-Cas systems were first considered as the adaptive immune system of bacteria (Garneau et al., 2010; Magadán et al., 2012) and in streptococci, *S. thermophilus* systems were among the best described and studied for many years. This species belongs to the large Salivarius group (Facklam, 2002). It is a thermophilic lactic acid bacteria used in the food industry in combination with *Lactobacillus* spp. in order to produce dairy products, like cheese or yogurts (Hols et al., 2005). The choice of strains used for dairy product manufacturing is essential as susceptibility to virulent phages can impair milk fermentation and lead to significant economic losses. Dairy industries had to adapt production techniques and select for bacteriophages-insensitive mutants (BIMs). Therefore, various typing methods have been developed to characterize them. Pulse-field gel electrophoresis (PFGE) was the first method used (Boutrou et al., 1995) and then, Random Amplification Polymorphic DNA (RAPD) was set up (Moschetti et al., 1998). More recently, four CRISPR-Cas systems were described in *S. thermophilus* and their analyses were proposed as a new typing method. CRISPR1 locus (type II-A) is ubiquitous, CRISPR2 (type III-A) and CRISPR3 (type II-A) loci are present in about 40% of strain (Horvath et al., 2008). The last locus, CRISPR4, is a type I-E system and is present in few strains (Horvath and Barrangou, 2010). Only CRISPR1 and CRISPR3 loci seem to be active, as they can acquire new spacers (Barrangou et al., 2013). The main advantage of CRISPR-typing in *S. thermophilus* is the presence of four different CRISPR loci, which allows better discrimination between isolates (Barrangou and Dudley, 2016). With this technique, producers can have an idea of meetings between phages and bacteria, and adapt strains used for milk fermentation. Other streptococcal species were investigated in order to search for CRISPR loci close to those of *S. thermophilus*. Results have shown that a homolog of CRISPR3 seems to be present in most streptococci species like *S. agalactiae*, *S. mutans* or *S. pyogenes*, instead of CRISPR1, whose homologs are found in only a few species, like *Streptococcus vestibularis* and *S. suis*. Homologs of the CRISPR2 system are more rare (Horvath et al., 2008). Tools have now been developed to find CRISPR loci in isolates and with the whole genome sequencing of bacteria, databases have been constituted to reference known systems (Grissa et al., 2007b; Couvin et al., 2018).

Global analysis of CRISPR-Cas systems, and particularly the spacer content, can thus provide information on species classification but also on their evolution. Previous studies have shown that old spacers, at the trailer end, are conserved between strains whereas new ones, at the leader end, are more variable (Barrangou and van der Oost, 2012). For these reasons, CRISPR loci analysis has been proposed as a promising new typing tool for many bacteria and particularly streptococci (Karimi et al., 2018). It is already used in many species, particularly in *Mycobacterium tuberculosis* (Brudey et al., 2006; Zhang et al., 2010) or in *Yersinia pestis* (Pourcel et al., 2005; Cui et al., 2008) and it could be useful for streptococci.

**FIGURE 1:**
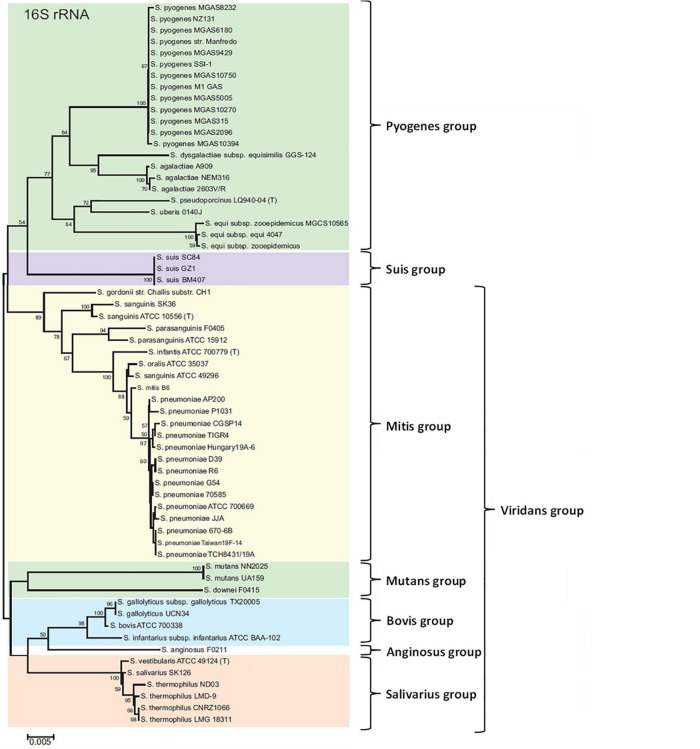
Seven groups formed from a neighbor-joining tree based on 16S rRNA gene sequences of *Streptococcus*. The numbers at the nodes indicate the values of bootstrap statistics after 2,000 replications, and values below 50% are not shown. Bars at 0.005% estimated sequence divergence. Adapted from Thompson et al. (2013).

As the *Streptococcus* genus comprises many species, that could contain one or more CRISPR-Cas systems, the aim of this review was to summarize knowledge about these systems and particularly their roles and applications in the well characterized pathogenic *Streptococcus* species. The presence and/or the types of CRISPR-Cas systems have been determined using the CRISPRCasfinder database, available at https://crisprcas.i2bc.paris-saclay.fr/ (Grissa et al., 2007a). The database contains more than 26,000 bacterial genomes, including 820 of streptococci, corresponding to 50 different species. Some differences could exist between results obtained with the CRISPRCasfinder database and data in the literature, in the subtypes of CRISPR-Cas systems and their distribution in the population. Indeed, this tool is based on a bioinformatical genome analysis and not on CRISPR-Cas systems functionality. Information concerning the distribution of CRISPR-Cas systems, along with the habitat and the pathogenicity of each *Streptococcus* species is resumed in Table 1. For more details in this review, we have chosen to focus on human pathogenic streptococci for which CRISPR-Cas systems have been already studied and described in literature (Table 2). This review was organized following the 16S rRNA classification described by Thompson et al. (2013) (Figure 1).

**TABLE 1:** Distribution of CRISPR-Cas systems in streptococci, with habitat and pathogenicity of all these species, based on CRISPRCasfinder analysis, available at https://crisprcas.i2bc.paris-saclay.fr/MainDb/StrainList (Grissa et al., 2007a).

Species	CRISPR-Cas systems (prevalence)	Habitat	Pathogenicity	References
*S. acidominimus*	Absence	Bovine vagina, calves skin, raw milk	– Bovine infection – Rarely pathogen in humans	Smith and Sherman, 1939; Wu et al., 2014
*S. agalactiae*	II-A (ubiquitous), I-C (some)	Human and animal microbiota (gastro-intestinal and genito-urinary tracts)	– Maternofetal infections – Invasive infections in elderly or immunocompromised people (bacteriemia, arthritis, meningitis) – Bovine mastitis and farm fish infections	Nocard and Mollereau, 1887; Spellerberg, 2000; High et al., 2005; Evans et al., 2009
*S. anginosus*	II-A (most), II-C (most), I-C	Human microbiota (oral cavity, upper respiratory, gastro-intestinal and genito-urinary tracts)	– Invasive infections [bacteriemia (often), endocarditis, abscesses (rare)]	Clarridge et al., 2001
*S. australis*	I-E	Human oral cavity	– Rare	Willcox et al., 2001
*S. canis*	II-A, I-C	Dog and cat skin and mucous membranes	– Invasive infections in mammals (abortion, septicemia) – Rare in humans	Devriese et al., 1986; Galpérine et al., 2007
*S. constellatus*	Absence	Human microbiota (oral cavity, upper respiratory, gastro-intestinal and genito-urinary tracts)	– Invasive infections (bacteriemia, endocarditis, deep abscesses (in upper body causing pulmonary exacerbations) – Periodontitis	Clarridge et al., 2001; Rams et al., 2014
*S. cristatus*	III-A, I-C	Human oral cavity and throat	– Rare	Handley et al., 1991
*S. dysgalactiae*	I-C (most), II-A (most)	Gastro-intestinal and genito-urinary tracts of humans and animals	– Skin and soft tissues infections – Pharyngitis – Bacteriemia – Bovine mastitis	Calvinho et al., 1998; Hughes et al., 2009a
*S. equi*	I-C (most), II-A (some)	Equine upper respiratory tract	– Pulmonary infections and abscesses	Sweeney et al., 2005
*S. equinus*	II-A, II-C, III-A	Animal and human gastro-intestinal tract	– Rare	Pompilio et al., 2019
*S. ferus*	Absence	Rodents oral cavity	– Rats dental infections (rare)	Freedman et al., 1982
*S. gallolyticus*	II-A, II-C	Human and animal gastro-intestinal tract	– Endocarditis – Associated with colorectal cancer	Rusniok et al., 2010; Boleij et al., 2011
*S. gordonii*	II-A, II-C	Human oral cavity	– Endocarditis (rare)	Xiong et al., 2008
*S. gwangjuense*	II-A	Dental cavity	– Rare dental infections	Park et al., 2019
*S. halotolerans*	I-C	Upper respiratory tract of marmot	– Unknown	Niu et al., 2016a
*S. himalayensis*	absence	Upper respiratory tract of marmot	– Unknown	Niu et al., 2017
*S. iniae*	II-A	Water, environment	– Severe infections in aquatic animals like dolphins or farm fish – Rare in humans	Goh et al., 1998; Zhang et al., 2014
*S. infantarius*	II-C, II-A	Human and animal gastro-intestinal tract	– Digestive infection and colorectal cancer association	Kaindi et al., 2018
*S. intermedius*	II-A, II-C	Human microbiota (oral cavity, upper respiratory, gastro-intestinal and genito-urinary tracts)	– Periodontitis – Invasive infections [bacteriemia, endocarditis, abscesses (brain)]	Clarridge et al., 2001; Rams et al., 2014
*S. koreensis*	Absence	Human oral cavity	– Periodontitis (rare)	Lim et al., 2019b
*S. lutetiensis*	II-C, II-A	Human and animal gastro-intestinal tract	– Digestive infections – Endocarditis (rare)	Waisberg et al., 2002
*S. macedonicus*	II-C	Thermophilic, fermentative bacteria, alimentary products	– Non-pathogenic	
*S. marmotae*	Absence	Lower respiratory tract of marmot	– Unknown	Niu et al., 2016b
*S. merionis*	I-C	Gastro-intestinal tract of Mongolian mice	– Unknown	Tappe et al., 2009
*S. mitis*	III-B (rare), II-C (rare)	Human oral cavity and upper respiratory tract	– Endocarditis and bacteriemia in immunocompromised people	Mitchell, 2011
*S. mutans*	II-A (most), II-C (some), I-C (most), I-E (some)	Human oral cavity, upper respiratory and gastro-intestinal tracts	– Dental infections – Endocarditis and bacteriemia in immunocompromised people	Ullman et al., 1988; Forssten et al., 2010
*S. oralis*	II-A, III-A	Human oral cavity	– Endocarditis and bacteriemia in immunocompromised people	Beighton et al., 1994
*S. pantholopis*	II-A, I-E	Gastro-intestinal tract of Tibetan antelopes	– Unknown	Bai et al., 2016
*S. parasanguinis*	I-C	Human oral cavity, dental plaque	– Dental infection (rare) – Endocarditis (rare)	Garnett et al., 2012
*S. parauberis*	absence	Animal gastro-intestinal tract	– Bovine mastitis – Infections in farm fish	Williams and Collins, 1990; Nho et al., 2011
*S. pasteurianus*	II-C, II-A	Human and animal gastro-intestinal tract	– Rare	Sturt et al., 2010
*S. periodonticum*	I-C	Oral cavity, dental plaque	– Gingival inflammation (rare)	Lim et al., 2019a
*S. pluranimalium*	Absence	Animal microbiota	– Bovine mastitis – Brain abscesses and septicemia in humans (rare)	Pan et al., 2018; Duriseti and Fleisher, 2019
*S. pneumoniae*	Absence	Human respiratory tract	– Pneumonia, otitis, bacteriemia, meningitis	Musher, 1992
*S. porcinus*	I-C, II-A	Pig upper respiratory tract	– Pig infections – Female genito-urinary tract infections (rare)	Collins et al., 1984; Facklam et al., 1995
*S. pseudopneumoniae*	Absence	Human respiratory tract	– Pneumonia	Mohammadi and Dhanashree, 2012
*S. pseudoporcinus*	II-A	Female genito urinary tract	– Female genito-urinary tract infections (rare)	Bekal et al., 2006
*S. pyogenes*	II-A (most), I-C (some)	Human microbiota (skin, upper respiratory tract)	– Superficial skin disorders (impetigo) – Upper respiratory tract infections – Invasive infections (bacteriemia, necrotizing fasciitis)	Cunningham, 2000
*S. ratti*	II-A, I-E	Oral cavity, dental plaque of humans and rats	– Rare	Garrett et al., 2020
*S. respiraculi*	II-A	Upper respiratory tract of marmot	– Unknown	Niu et al., 2018
*S. ruminantium*	II-C	Pig microbiota	– Unknown	Tohya et al., 2018
*S. salivarius*	II-C, II-A, III-A	Human oral cavity, skin, respiratory, gastro-intestinal and genito-urinary tracts	– Invasive infections like bacteriemia in immunocompromised people	Corredoira et al., 2005
*S. sanguinis*	II-A, III-A	Human oral cavity, dental plaque	– Endocarditis and bacteriemia in immunocompromised people (rare)	Douglas et al., 1993
*S. sobrinus*	I-E	Human oral cavity, dental plaque	– Dental infections (dental caries)	Conrads et al., 2014
*S. suis*	II-A, II-C	Pig respiratory and genito-urinary tracts	– Pig infections – Invasive infections in humans (rare)	Lun et al., 2007; Gottschalk and Segura, 2019
*S. thermophilus*	III-A (most), II-C (most), II-A (most), I-E (some)	Thermophilic, fermentative bacteria, alimentary products	– Non-pathogenic	
*S. troglodytae*	II-C, I-E	Oral cavity in monkeys	– Unknown	Okamoto et al., 2016
*S. uberis*	II-C, II-A	Environment	– Bovine mastitis	Zadoks et al., 2001
*S. urinalis*	Absence	Human genito-urinary tract	– Rare	Collins et al., 2000
*S. vestibularis*	II-C	Human oral cavity and respiratory tract	– Rare invasive infections (bacteriemia, endocarditis after dental intervention)	Simsek et al., 2008

**TABLE 2:** Roles and applications derived from CRISPR-Cas systems of pathogenic streptococci, described in literature.

		Roles and applications					
	CRISPR systems (presence)	Defense system against MGEs	Typing method	Species evolution	Regulation	Antibiotics susceptibility	Genome editing
*S. agalactiae*	II-A (ubiquitous), I-C (some)	Bolotin et al., 2005	Lier et al., 2015	Beauruelle et al., 2017	Lopez-Sanchez et al., 2012		
*S. anginosus*	II-A (most), II-C (most), I-C	Olson et al., 2013		Pride et al., 2011	Bauer et al., 2020		
*S. mutans*	II-A (most), II-C (some), I-C (most), I-E (some)	Van der Ploeg, 2009		Maruyama et al., 2009			
*S. pyogenes*	II-A (most), I-C (some)	Deltcheva et al., 2011	Zheng et al., 2015		Bikard et al., 2013	Zheng et al., 2014	Cong and Zhang, 2015

## Pyogenes Group

### Streptococcus agalactiae

*Streptococcus agalactiae*, or group B *Streptococcus*, was originally described in bovine mastitis (Nocard and Mollereau, 1887; Wanger and Dunny, 1987). Since the 1960’s, it has appeared in human medicine as a major pathogen in maternofetal infections (Bergqvist and Hurvell, 1970; McCracken, 1973; Raabe and Shane, 2019). This species belongs to the commensal microbiota and colonizes the gastro-intestinal and genito-urinary tracts in about 30% of people (Van der Mee-Marquet et al., 2008). Today, it is still the leading cause of neonatal infections in developed countries (Verani and Schrag, 2010; Furfaro et al., 2018). Moreover, it is also increasingly involved in adult infections, especially in elderly and immunocompromised people, causing bacteremia, meningitis, arthritis, etc. (Farley and Strasbaugh, 2001; High et al., 2005).

The typing of *S. agalactiae* isolates is important as pathogenesis and virulence are different according to strains, especially in infants (Poyart et al., 2008). Firstly, ten serotypes have been described by analyzing capsular polysaccharides, and serotype III has been identified as the most associated with neonatal infections. As the discrimination with this method was not sufficient, multilocus sequence typing (MLST), which consists in sequencing seven housekeeping genes, has been developed (Jones et al., 2003). MLST is the current reference method used to distinguish *S. agalactiae* isolates, and more than 1,600 different sequence types (ST) are now described. This method has a good discriminating power but, with the discovery and research on CRISPR-Cas systems, recent studies propose CRISPR-typing as a promising alternative method (Beauruelle et al., 2017, 2021).

Two CRISPR loci have been identified in *S. agalactiae*, the CRISPR1 locus, which is a type II-A system, and the CRISPR2 locus, which belongs to type I-C systems, based on the current classification (Table 1; Lopez-Sanchez et al., 2012). The first one is ubiquitous and dynamic, whereas the second is present in a minority of strains. Firstly, the sequencing of the CRISPR1 locus has shown that direct repeats were usually highly conserved between strains, whereas the terminal repeat has many variants, which defined different groups. These groups correlate well with the phylogenetic lineages determined using the MLST method (Lier et al., 2015). Moreover, the analysis of the spacers content has shown a high degree of polymorphism which could probably lead to very precise classification. In order to confirm this hypothesis, the CRISPR-typing method has been compared to existing techniques. It has been shown that CRISPR-typing correlates very well with the MLST method and could even be more precise thanks to the analysis of the most recent spacers and according to that, identified subgroups within a same ST (Lier et al., 2015). Other studies have shown the high discriminating power of CRISPR-typing. An initial study, which followed the vaginal carriage of *S. agalactiae* in women for 11 years, demonstrated that CRISPR-typing correlates well with the results obtained by the MLST method and gives more information about colonizing strains, such as MGEs encountered in the vaginal environment (Beauruelle et al., 2017). Indeed, CRISPR-typing makes it possible to classify strains into ST like MLST, but it is more precise because it highlights differences in strains within the same ST according to the spacers present in the locus. Another study, exploring the diversity of strains in vaginal swabs of pregnant women by CRISPR-typing, demonstrated that some women carried isolates belonging to the same phylogenetic group (sharing the same CRISPR1 locus), others carried homogeneous population of isolates with few differences at the CRISPR locus, and some women are colonized at the vaginal level by isolates belonging to different phylogenetic groups (Beauruelle et al., 2018).

Thus, the *S. agalactiae* CRISPR1-Cas system appears to be a useful tool for typing isolates but also for providing information about the evolution of this species (Table 2). It has also been shown that it could be involved in the differences of virulence observed between strains by integrating or not integrating new spacers from MGEs. Indeed, virulence genes are frequently acquired by horizontal gene transfer (Koonin et al., 2001) and CRISPR-Cas systems can be a barrier to this transfer. The *S. agalactiae* genome is divided in a “core genome,” which is the conserved part of the genome shared by all the strains, and a “dispensable genome,” which is a variable part and can correspond to horizontal gene acquisition (Tettelin et al., 2005). According to the CRISPR-Cas system’s efficiency and its spacers contents, strains are more or less susceptible to invaders and consequently to the acquisition of new genes. However, these genes can improve the pathogenicity and the survival of bacteria, as they can correspond to resistance or virulence genes. Differences have been highlighted according to ST. For example, strains belonging to ST17, considered as hypervirulent because more involved in neonatal infections, have less spacers than other STs (Lier et al., 2015). More recently, specificity in the locus organization has been highlighted for these strains and the system’s functionality has been explored (Pastuszka et al., 2021). Thanks to its CRISPR1-Cas system, *S. agalactiae* can not only defend itself against invaders but also regulate its own genome. It was shown that spacer diversity is important and some of them target MGEs integrated in the bacterial genome, in order to regulate what is called the “mobilome” of *S. agalactiae* (Lopez-Sanchez et al., 2012). Other spacers target genes from the core genome, and in order to differentiate between self- and non-self-targeting, the PAM plays an essential role and allows fine regulation (Mojica et al., 2009).

In *S. agalactiae*, like in most streptococci, CRISPR-Cas systems have many important roles and their analyses can provide a lot of information about species and the differences observed between strains.

### Streptococcus pyogenes

*Streptococcus pyogenes*, according to the Lancefield classification, is a group A *Streptococcus* involved in human pathology. It is a strictly human bacteria, which belongs to the commensal microbiota of the skin, or may be found in the upper respiratory tract, according to age (Hartley et al., 1945; DeMuri and Wald, 2014). Initially, it was implicated in mild illnesses like impetigo, a superficial skin disorder, or infections of the upper respiratory tract, such as pharyngitis (Stevens and Bryant, 2016). Since the 1980’s, an increased incidence of invasive infections, like bacteremia or necrotizing fasciitis, has been highlighted (Carapetis et al., 2005; Lamagni et al., 2008).

Two CRISPR loci have been described in *S. pyogenes*, CRISPR1 and CRISPR2 (Nozawa et al., 2011), belonging, respectively, to type II-A and type I-C systems (Table 1; Marraffini, 2016). CRISPR analysis is an element which provides a better understanding of the evolution of species because it reflects meetings of bacteria over time and their adaptation to them (Table 2; Le Rhun et al., 2019). Among streptococci, *S. pyogenes* possesses less spacers than other species, and some strains have completely lost their CRISPR loci. These findings have demonstrated that the strains, which have no or few spacers, contain more virulence factors acquired from MGEs (Nozawa et al., 2011). The loss of a CRISPR-Cas system could favorize an adaptation leading to a better bacterial permeability, allowing to acquire new virulence factors in order to improve their pathogenicity. But CRISPR-Cas systems are still active because it was shown that some strains are resistant to phages infection, thanks to a recent spacer acquisition. The presence of a spacer with homology with a phage sequence conferred resistance against prophage insertion to these strains. CRISPR-Cas system protects bacteria from phages lysis but this can also lead to a potential decrease in virulence. Indeed, it has been shown that up to 14% of the *S. pyogenes* genome is encoded by prophage insertions, often providing them virulence factors, like exotoxins, for example (Fischetti, 2007), leading to the scarlet fever (Yamada et al., 2019). The balance between defense mechanisms protecting bacteria integrity, and invasion permission in order to be more virulent, is very important and changing over time, leading to species evolution.

*Streptococcus pyogenes* is a major pathogen involved in severe invasive infections. Methods used to characterize species have been developed in order to better understand virulence differences observed between isolates. Firstly, sera agglutination was used to serotype isolates, based on antigenic properties. M protein, encoded by *emm* gene, a surface protein and a virulence factor carried by the strain was used initially. A few years later, it was decided to sequence a part of this gene in order to establish a more precise classification (Beall et al., 2000; McMillan et al., 2013b). *Emm*-typing is currently the reference method, and more than 200 *emm*-types have been described (McMillan et al., 2013a; Sanderson-Smith et al., 2014). *Emm1* was the most prevalent type (Hoe et al., 1999), but since the 2000s other types, like *emm28* and *emm89*, are emerging worldwide (O’Brien et al., 2002; Ikebe et al., 2007; Luca-Harari et al., 2009). All *emm1*, *emm12*, and *emm28* strains possess two CRISPR loci and *emm3* strains have no CRISPR2 locus. Analyzing the composition of CRISPR loci, has enabled to show relations between spacer content and *emm*-types (Zheng et al., 2015). Indeed, they have highlighted that most spacers are *emm*-type specific and so, they propose CRISPR sequencing as a new typing method. The analysis of all the CRISPR loci present in a strain, or of each locus alone, have a higher Wallace coefficient than the *emm*-typing, proving the good congruence of both methods (Severiano et al., 2011). These results place the CRISPR-typing method as a good alternative to characterize *S. pyogenes* strains, and a better technique to differentiate isolates classified in the same *emm*-type. As previously mentioned, sequencing only one locus may not be sufficient to differentiate subtypes, and a combination of both loci is necessary. For example, the CRISPR1 locus analysis only is not able to differentiate *emm3* and *emm4* types, and the sequencing of the CRISPR2 locus is essential. However, *emm*-typing will remain useful, particularly for strains which do not contain CRISPR loci. It has been established that CRISPR loci of *S. pyogenes* acquire few spacers compared to other streptococci, which places this typing method as a good tool for this species. Indeed, there is not much spacer deletion or spacer insertion in loci, so less variability between regions in the world, giving stability and reliability to CRISPR-typing (Nozawa et al., 2011).

Studies have also shown that analyzing *S. pyogenes* CRISPR arrays can provide additional information concerning isolates, particularly reflecting encounters and evolution of species. For example, it may predict macrolide susceptibility (Zheng et al., 2014). This observation is in relation with the adaptive immune defense role of the system. Strains with a lot of spacers are generally more susceptible to erythromycin. Indeed, the resistance gene to this antibiotic is carried by a MGE, which will be cleaved in the event of sequence homology between this invader DNA and spacers present in the locus. Fighting against invasion by MGEs also prevents bacteria from acquiring new genes, which may support resistance or virulence genes.

However and rapidly, the CRISPR-Cas9 system of *S. pyogenes* has been used in genetic engineering because of its ease of use (Cong and Zhang, 2015) and the Nobel Prize was awarded for this application in 2020. Many discoveries have enabled the development of this application and particularly the demonstration of the role and importance of tracrRNA (Deltcheva et al., 2011). This extensive use has made CRISPR-Cas systems popular and interesting for many people. Nevertheless, more than its principal use in genome editing, CRISPR-Cas systems are very important in *S. pyogenes* and their analyses are promising in strain classification and for understanding the evolution of the species.

### Other Members

The pyogenes group comprises other species that can be involved in human pathologies, among which *Streptococcus dysgalactiae, Streptococcus pseudoporcinus, Streptococcus porcinus, Streptococcus equi*, etc. These species can carry a type I-C and/or a type II-A CRISPR-Cas system, which are the most frequent in Streptococci, but no data is currently available about their role (Table 1).

## Suis Group

### Streptococcus suis

*Streptococcus suis* can lead to invasive infections in humans, especially in people working with pigs (Hughes et al., 2009b). According to the CRISPR finding tool, strains can carry type II-A and/or type II-C CRISPR-Cas systems (Table 1). As demonstrated in a study highlighting defense systems against invading DNA in this species, three CRISPR-Cas loci were identified (Okura et al., 2017).

## Viridans Group

The viridans group is composed of five subgroups: anginosus group, bovis group, mitis group, mutans group and salivarius group.

### Anginosus (or Milleri) Group

#### Streptococcus anginosus

*S. anginosus* is part of the human microbiota and is found in the oral cavity, the gastro-intestinal and the genito-urinary tracts (Whiley et al., 1992). Since many years, it has been increasingly involved in deep infections, like bacteremia and abscesses (Reißmann et al., 2010; Siegman-Igra et al., 2012), and is therefore considered as an important pathogen.

CRISPR loci have been described in *S. anginosus* previously. Most strains carry a type II-A and/or a type II-C system (Table 1). Few strains possess a type I-C system. Similarities have been demonstrated with systems found in other oral streptococci, like *S. mutans* or *S. sanguinis*, which suggest horizontal gene transfer between species (Olson et al., 2013). Sequence homologies between spacers and foreign genetic elements have been highlighted for all these CRISPR-Cas systems (Table 2).

Within this species, strains can be alpha-hemolytic, beta-hemolytic or non-hemolytic (Jensen et al., 2013). Beta-hemolysis of some strains is due to the presence of a virulence factor encoded by *sag* genes (Asam et al., 2013). This hemolysin seems to be acquired from a *S. pyogenes* genetic locus, encoding a bacteriocin-like peptide, the streptolysin S (Nizet et al., 2000). An inverse correlation has been shown between the presence of a CRISPR-Cas system and the presence of *sag* genes in *S. anginosus* isolates (Bauer et al., 2020). Indeed, the presence of a CRISPR locus, and especially its spacer content, gives the strain the ability to fight against integration in its genome of foreign DNA pieces if homology exists. This avoids the transfer and acquisition of genetic elements like virulence factors from MGEs, such as hemolysin for example. Thus, it is possible to classify strains and predict their virulence by analyzing their CRISPR arrays. CRISPR-typing has also been used in a study to follow the presence of streptococci in the salivary microbiota, in order to visualize differences, over time, in a single subject and between subjects. The aim of this study was to better understand the evolution of bacteria and their encounters with viruses or other MGEs (Pride et al., 2011). CRISPR arrays analysis seems to be a promising typing tool in this species, but must be continued in order to enhance knowledge.

#### Other Members

The large anginosus group, which is also called the milleri group, includes two other important species in human pathology. *Streptococcus constellatus* and *Streptococcus intermedius* belong to the human microbiota and can be involved in severe invasive infections like bacteremia, endocarditis, or deep abscesses (Tran et al., 2008; Chrastek et al., 2020). Concerning the presence of CRISPR-Cas systems, two types can be found in *S. intermedius*, type II-A and type II-C, like in *S. anginosus* but they are less well studied (Ben Zakour et al., 2012). However, no CRISPR-Cas system has been identified in *S. constellatus* (Table 1).

### Bovis Group

Species of this group are particularly associated with digestive infections and colorectal cancers (Boleij et al., 2011; Kaindi et al., 2018) and can be responsible for endocarditis (Rusniok et al., 2010). Moreover, thanks to the CRISPR finder tool, two CRISPR-Cas systems can generally be found, a type II-A and a type II-C (Table 1). Loci organization of CRISPR-Cas systems has been analyzed in *S. gallolyticus* but it is no longer studied (Lin et al., 2011).

### Mitis Group

The mitis group comprises many species including the well-known human pathogen *Streptococcus pneumoniae* and many other commensal species belonging to the human oral cavity and that can lead to invasive infections, especially in immunocompromised people (Mitchell, 2011; Weiser et al., 2018; Basaranoglu et al., 2019). A wide range of different CRISPR-Cas systems can be identified depending on the species (Table 1).

#### Streptococcus mitis

According to the most recent version of the CRISPR finder tool, it has been shown that *Streptococcus mitis* strains can carry type II-C or type III-B CRISPR-Cas systems, but it seems extremely rare. It has been described that some strains carry CRISPR-like sequences, but they are not well characterized (Maeda et al., 2011). Indeed, these sequences share similarities with CRISPR loci, suggesting that strains which carry them could share a common ancestor.

#### Streptococcus pneumoniae

**FIGURE 2:**
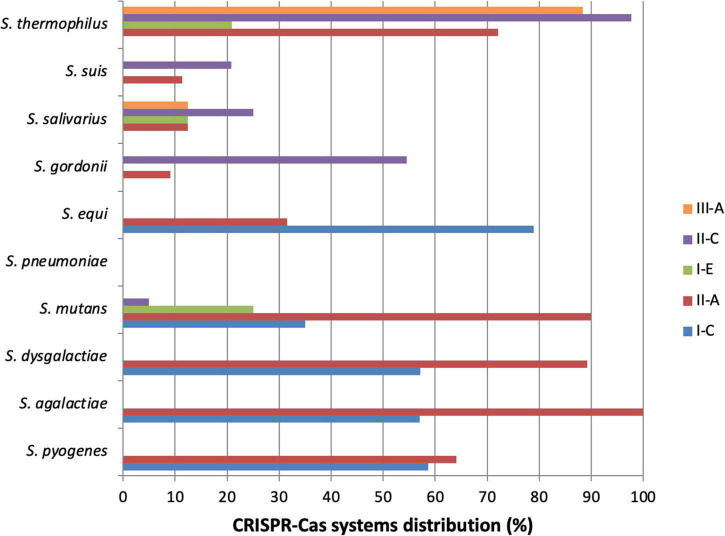
Distribution of CRISPR-Cas systems in *Streptococcus* species. This graph represents the distribution of CRISPR-Cas systems in some *Streptococcus* species. It is based on the data available on “CRISPR finder” online, considering the tool limits. Particularly for *S. thermophilus*, it is important to notice that type II-C CRISPR-Cas systems found are actually type II-A. Only species of which at least 10 strains have been sequenced and analyzed are represented. *S. thermophilus* (*n* = 43), *S. suis* (*n* = 53), *S. salivarius* (*n* = 16), *S. gordonii* (*n* = 11), *S. equi* (*n* = 19), *S. pneumoniae* (*n* = 141), *S. mutans* (*n* = 20), *S. dysgalactiae* (*n* = 28), *S. agalactiae* (*n* = 121), *S. pyogenes* (*n* = 220).

*Streptococcus pneumoniae* belongs to the human respiratory tract and is an important pathogen in human medicine. Indeed, it can be responsible for many common infections like otitis or pneumonia, but also for more invasive infections, like bacteremia and meningitis (Musher, 1992; Weiser et al., 2018). *S. pneumoniae* strains generally do not possess CRISPR locus (Table 1 and Figure 2), but can sometimes carry a remnant system. Indeed, homologous sequences to those coding CRISPR-Cas systems have been found, but no functional CRISPR-Cas system has been actually described in this species. It is supposed that *S. pneumoniae* possessed a CRISPR-Cas system a few years ago, but for some reason it lost it. Several explanations are plausible. The most likely hypothesis is that *S. pneumonia*e is a naturally competent bacteria making numerous genetic exchanges via horizontal gene transfers in order to adapt to its environment. CRISPR interference can be an obstacle to these transfers, so the loss of a CRISPR-Cas system can be beneficial for species evolution. Other hypotheses can be that *S. pneumoniae* is not often in contact with phages, so it is not useful, or because the system is too inordinate for the growth and survival of bacteria (Bikard et al., 2012).

### Mutans Group

The mutans group is composed of several streptococci but CRISPR-Cas systems have been already described and studied in *S. mutans* only.

#### Streptococcus mutans

*Streptococcus mutans* belongs to the human buccal cavity. Like other buccal streptococci, it is involved in dental infections, and particularly in dental caries (Hamada and Slade, 1980). Because of the increasing antibiotic resistance in bacteria, research focused on phage therapy to eliminate *S. mutans* from the dental plaque of people prone to dental caries. Lytic phages have been isolated (Delisle and Rostkowski, 1993) but *S. mutans* has already developed resistance to some of them. Studying the origin of these resistances led to the discovery of CRISPR loci in this species (Shibata et al., 2009; Serbanescu et al., 2015).

Two CRISPR loci have been described in *S. mutans* (Haft et al., 2005). The CRISPR1 locus is a type II-A system and the CRISPR2 locus is a type I-C system (Table 1). Spacers of these two loci have been shown to share similarities with phage sequences (Van der Ploeg, 2009). Moreover, strains resistant to some phages have specific spacers that sensitive strains do not have. The CRISPR1 locus (Mosterd and Moineau, 2020) is more present than the CRISPR2 in strains but none is ubiquitous. A great variability in their spacer content has been highlighted and shown that they are a good tool for *S. mutans* typing, especially the CRISPR2 locus, which is more variable and best reflects the evolution of this species (Table 2; Maruyama et al., 2009). Since the description of these two systems, two other CRISPR-Cas systems have been integrated in CRISPR databases, which are a type II-C system and a type I-E system. The typing of *S. mutans* strains is interesting in human medicine. A very high number of STs have been described, showing that this species is very diverse (Nakano et al., 2007). This diversity between isolates was confirmed using the CRISPR arrays analysis. Its information provides insight into the bacteria encounters and resistance genes that they carried. In another study, it was also shown that *S. mutans* CRISPR-Cas systems were involved in strain virulence. Indeed, it was shown that *cas* genes were more transcribed when strains were submitted to stress and also during biofilm formation, which indicates their implication for bacteria persistence in its environment (Louwen et al., 2014). A more recent study analyzed the clinical strains of *S. mutans*, isolated from early childhood dental caries. They found that the spacer content of the CRISPR1 and CRISPR2 loci was very diverse (Chen et al., 2017). They also showed that strains carrying a CRISPR-Cas system were more virulent than strains without a CRISPR-Cas system, because they had a better ability to produce extracellular polysaccharides and form biofilm on teeth surfaces. This was supported by another study, which showed that strains deleted for *cas3* (type I CRISPR-Cas system) were less virulent and produced a finer biofilm than wild type strains. They also showed that *cas3* deleted mutants were more susceptible to fluoride, which can be a treatment used to reduce biofilm formation (Tang et al., 2019).

Further studies are required for this species in order to better understand *S. mutans* strains evolution and perhaps adapt treatment strategies.

### Salivarius Group

Species from this group like *Streptococcus vestibularis* or *Streptococcus salivarius* belong to the human microbiota and are usually found in the oral cavity. They can be involved in bacteremia in immunocompromised people, but it is still rare (Corredoira et al., 2005). According to the CRISPR finder tool, strains can carry type II-A, type II-C or type III-A CRISPR-Cas systems (Table 1), and sequence similarities between spacers and extrachromosomal elements have been shown (Bolotin et al., 2005).

*Streptococcus thermophilus* is also a member of this group. It is not a pathogen for humans or animals, so is not detailed in this review, but cannot be ignored when referring to CRISPR-Cas systems, because it was the first studied *Streptococcus*.

## Discussion

Streptococci are a wide group of bacteria and an important genus in human and animal medicine (Lamagni et al., 2008; Haas and Grenier, 2018; Raabe and Shane, 2019). For each species, differences in evolution and virulence have been highlighted, depending on the isolated strain. Since their discovery, research on CRISPR-Cas systems has shown that sequencing CRISPR loci can provide numerous isolate information, about their virulence, foreign MGEs, adaptation and evolution (Bolotin et al., 2005; Horvath et al., 2008; Mojica et al., 2009). A great majority of streptococci contain one or more CRISPR loci, or at least sequences which have similarities with CRISPR loci but cannot be qualified as such (Moore et al., 2008). Only two types of CRISPR-Cas systems can be found for most bacterial species, but all three types can be found specifically for streptococci (Figure 2), and particularly the subtypes I-C, II-A and III-A (Louwen et al., 2014; Bauer et al., 2020). Subtype II-A is particularly present in streptococci, like in many commensal and pathogenic bacteria in humans (Chylinski et al., 2014). Thanks to existing databases presence of CRISPR-Cas systems is relatively well known in streptococci. To improve knowledges, the exact distribution and the study of the genes conservation inside species, and between species that contain the same type of CRISPR-Cas system should be of interest. If strong identities are found, this could expand our knowledge of the function of the system in one species to other species for which little data are currently available. Large-scale bioinformatic analyses will be necessary to complete this work.

One of the most developed applications of the CRISPR-Cas systems is their use in genome editing. Because of the simple multi-domain single-protein effector action, class 2 CRISPR-Cas systems are the most extensively used for genome editing. First applications were derived from Cas9 endonucleases, but Cas12 endonucleases were rapidly used (Anzalone et al., 2020). Cas13 is also used, but it preferentially targets RNA. Some differences exist between these nucleases, such as the nucleic acid cleavage site, and the choice of whether to use one or the other depends on the desired result. With these nucleases, double stranded or single stranded DNA can be cut or modified, depending on the Cas variant used. This plasticity and this wide range of possibilities means that CRISPR-Cas derivatives are very promising tools for treating some diseases by gene therapy. Techniques have now been improved and multiplex gene editing is almost available. Cas proteins have already been modified in order to improve the affinity with a targeted DNA and recognize more variable PAM sequences. Techniques were also improved to limit off-target and be more precise in order to insert modifications in DNA sequences. Thanks to these applications, progress has been made in better understanding some diseases, by introducing mutations in genes and observing the consequences, in order to highlight the function of these genes and their implications (Wang et al., 2016). Research is ongoing for use in gene therapy, such as treating cystic fibrosis (Schwank et al., 2013) or beta-thalassemia (Xie et al., 2014). Work must be continued in order to improve use and answer all given queries before being able to cure diseases.

More than this important and great use, numerous studies have shown that analyzing CRISPR arrays can provide a lot of information about streptococci isolates. Its main role of immune defense system confers an ability to fight against MGEs via integrated DNA sequences as spacers into its locus and the analysis of the spacer contents shows an encounter of streptococci with these MGEs. CRISPR-Cas systems can regulate their ability to acquire, or not, new genes from other bacteria, phages or various MGEs, and so, to acquire, or not, virulence or resistance genes. This could partly explain the differences in virulence observed between strains. This virulence involvement has already been demonstrated for other bacterial species (Louwen et al., 2014; Ahmed et al., 2018). *Francisella novicida* uses its CRISPR-Cas system to repress the expression of a lipoprotein that will not be recognized by the host immune system, and thus progress in infections (Sampson and Weiss, 2014). *Pseudomonas aeruginosa* can also be cited as an example. It was established that the type I-F CRISPR-Cas system is able to repress some genes in order to escape the host defense immune system and reduce the amount of cytokines produced by the host (Li et al., 2016; Wiedenheft and Bondy-Denomy, 2017). With this review, we can see that this link between CRISPR-Cas systems and bacterial virulence exists also in streptococci, as proved with *S. pyogenes* (Nozawa et al., 2011), *S. mutans* (Chen et al., 2017) or *S. anginosus* (Bauer et al., 2020). A link has also been established between CRISPR-Cas systems and antibiotics resistance, as demonstrated for *S. pyogenes* and the macrolides susceptibility. Furthermore, with the increasing dissemination of antibiotics resistance genes, two new uses of CRISPR-Cas9 genome editing have been developed. The first one consists in programming Cas9 endonuclease to target and cleave essential genes of bacteria and, therefore, kill a targeted bacterial population (Bikard et al., 2014; Gomaa et al., 2014). The second consists in using a “dead” Cas9 (dCas9), which is able to bind to a targeted element but not cleave it. This binding could repress the gene transcription and could be interesting in targeting antibiotics resistance genes without killing bacteria (Bikard et al., 2013).

In addition, this review shows that, especially in streptococci but also in other bacteria, sequencing CRISPR loci is also a relatively easy way to characterize and classify isolates precisely. Indeed, many studies have demonstrated that using CRISPR as a typing method is a promising tool and must be developed (Pourcel et al., 2005; Brudey et al., 2006; Beauruelle et al., 2021). More than precisely classifying strains, it can give a lot of information about species evolution.

Finally, the adaptive immune role of CRISPR-Cas systems, providing bacteria with a strong protection against phages and other MGEs was first elucidated in a *Streptococcus* species, *S. thermophilus*. However, phages, stuck in a perpetual evolutionary arms race with bacteria, have developed different mechanisms to counter these bacterial defenses. The simplest way was to acquire mutations in the targeted sequences, until the discovery of small proteins, capable of antagonizing the CRISPR-Cas systems, the anti-CRISPR (Acr) proteins. The first Acrs were described in *Pseudomonas aeruginosa* prophages with efficacy against type I-E and I-F CRISPR-Cas systems (Bondy-Denomy et al., 2013). Indeed, Acrs have been frequently identified in temperate phages, genomic islands or prophages, suggesting that their presence would be necessary for these elements to be maintained in a bacterial genome carrying a CRISPR-Cas system. Streptococci may contain many prophages and, not surprisingly, bioinformatic tools can predict Acrs in their genomes, like in *S. pyogenes* and other streptococcal species carrying a type II-A CRISPR-Cas system (Eitzinger et al., 2020). Depending on the Acrs types, different stages of CRISPR-Cas systems can be blocked. In consequence, Acrs can have a more or less broad activity spectrum depending on the Cas proteins targeted, and the inhibitory mechanism involved. Since their discovery, Acr genes have been identified in many bacteria, with a wide range of activities, inhibiting diverse class 1 or class 2 systems. Some of them are even able to block the activity of SpCas9, the widely used genome-editing tool, especially Acrs described in *S. thermophilus* virulent phages (Hynes et al., 2017; Rauch et al., 2017). All these elements make Acrs a promising tool to control Cas9 activities in genome editing.

Many tools have been developed since the discovery of the CRISPR-Cas systems, but a lot of studies remain to be performed on streptococcal species that have not been explored yet, in order to improve our knowledge of these systems, and of their more recently discovered anti-systems, and definitely develop new applications.

## Author Contributions

CL, AP, and PL designed the study and wrote the manuscript. BL and LM provided critical feedback of the manuscript. All authors contributed to the article and approved the submitted version.

## Conflict of Interest

The authors declare that the research was conducted in the absence of any commercial or financial relationships that could be construed as a potential conflict of interest.

## Publisher’s Note

All claims expressed in this article are solely those of the authors and do not necessarily represent those of their affiliated organizations, or those of the publisher, the editors and the reviewers. Any product that may be evaluated in this article, or claim that may be made by its manufacturer, is not guaranteed or endorsed by the publisher.
